# The Signaling Pathways Involved in Chondrocyte Differentiation and Hypertrophic Differentiation

**DOI:** 10.1155/2016/2470351

**Published:** 2016-12-15

**Authors:** Jianmei Li, Shiwu Dong

**Affiliations:** Department of Biomedical Materials Science, School of Biomedical Engineering, Third Military Medical University, Chongqing 400038, China

## Abstract

Chondrocytes communicate with each other mainly via diffusible signals rather than direct cell-to-cell contact. The chondrogenic differentiation of mesenchymal stem cells (MSCs) is well regulated by the interactions of varieties of growth factors, cytokines, and signaling molecules. A number of critical signaling molecules have been identified to regulate the differentiation of chondrocyte from mesenchymal progenitor cells to their terminal maturation of hypertrophic chondrocytes, including bone morphogenetic proteins (BMPs), SRY-related high-mobility group-box gene 9 (Sox9), parathyroid hormone-related peptide (PTHrP), Indian hedgehog (Ihh), fibroblast growth factor receptor 3 (FGFR3), and *β*-catenin. Except for these molecules, other factors such as adenosine, O_2_ tension, and reactive oxygen species (ROS) also have a vital role in cartilage formation and chondrocyte maturation. Here, we outlined the complex transcriptional network and the function of key factors in this network that determine and regulate the genetic program of chondrogenesis and chondrocyte differentiation.

## 1. Introduction

Chondrocyte differentiation and hypertrophy are key events in bone formation. There are two processes in bone formation: one is intramembranous ossification, during which mesenchymal stem cells (MSCs) directly differentiate into osteoblasts. Irregular bones including the skull bone, clavicle, and part of the jaw are formed in this way. On the other side, the long bones and vertebrate skeleton are formed by another bone forming process of endochondral ossification. Simply put, endochondral ossification is a process that starts with the condensation of MSCs, which differentiate to chondrocyte and then form a cartilage template. Gradually, the cartilage template is replaced by bone minerals. MSCs during condensation will express Sox9, which is a pivotal regulatory factor in chondrogenesis [1]. And these cells differentiate into chondrocytes with two subpopulations: round, low proliferating chondrocytes at the distal ends of the condensation that continue to express Sox9 and high proliferating chondrocytes lying in columns towards the center that then undergo maturation [1–4]. During the process of maturation, chondrocytes in the central region of cartilage anlagen withdraw from the cell cycle and differentiate into prehypertrophic and hypertrophic chondrocytes with about 20-fold increase in volume [5]. Hypertrophic chondrocytes produce mineralized extracellular matrix, which is a template for the subsequent replacement by bone.

Surrounding the cartilage element is the perichondrium that consists of a layer of fibroblast-like cells. The cells in perichondrium will differentiate into osteoblasts to form the highly vascularized periosteum. And then blood vessels from the periosteum together with osteoblasts and osteoclasts invade the calcified matrix produced by hypertrophic chondrocytes, resulting in the replacement of mineralized cartilage by bone to form the primary ossification center. And the matrix will be further remodeled to form the cortical bone and the bone marrow cavity to provide a hematopoietic environment. Secondary ossification centers usually arise at the stage of postnatal development and locate at the distal ends of the long bone.

Gene expression varies with the different stage of endochondral ossification. The immature chondrocytes express the transcription factors Sox5, Sox6, and Sox9 and the structure proteins collagen type II, *α*1, aggrecan. All of those are the markers of chondrocyte differentiation. After that stage is chondrocyte prehypertrophy, which is marked by parathyroid hormone 1 receptor (Pth1r) and Indian hedgehog (Ihh) expression. Then, the stage goes into early hypertrophic chondrocytes that express collagen, type X, *α*1 (Col10a1). And subsequently the expression of Sox5, Sox6, Sox9, and Col2a1 was decreased. Finally, chondrocytes proceeded into late hypertrophic status with the expression of vascular endothelial growth factor A (VEGFA), matrix metalloproteinase 13 (MMP13), and osteopontin. These genes expressions herald the matrix being invaded by osteoblasts, osteoclasts, and endothelia cells and that means the cartilage templates are going to be replaced by bones. Studies have reported that the development of bones is dependent on the balance between chondrocyte proliferation and differentiation. Once the balance is disturbed, the length and stability of bones will be changed.

During the past years, large number of human chondrodysplasias and multitude of transgenic mice that show skeletal defects suggested the important role of chondrocyte differentiation in cartilage and bone development and showed us the basic biology of cartilage and bone. The fine-tuning of the chondrocyte maturation process will lead to various skeletal pathologies. So, it is necessary for us to make a thorough study about the mechanisms of chondrocyte differentiation and hypertrophy differentiation. Although a number of critical signaling and transcription factors have been identified playing an important role in regulating cartilage formation and chondrocyte differentiation from initial MSCs into mature terminal hypertrophic chondrocytes by amount of work both in vivo and in vitro, the molecular events of how the signals are translated into gene expression are still largely unknown, and our data about the mechanisms that regulate the initial steps of chondrogenesis are limited. In this review, we summarized the current knowledge about the relevant signaling pathways and transcription factors that regulate chondrocyte differentiation.

## 2. Sox9 Signaling

The transcription factor Sox9, which belongs to the family of “high-mobility group-box” transcription factors, is a pivotal transcription factor in the developing and maturation of cartilage. It is one of the earliest markers of condensing chondrocytes, which is expressed from multipotent skeletal progenitor stage and is maintained throughout life in permanent chondrocytes of healthy articular cartilage. However, the expression of Sox9 will be repressed in hypertrophic chondrocytes [6]. Studies indicated that Sox9 has a key role in the chondrogenic differentiation program and multiple signaling pathways regulate the expression and activity of Sox9 during chondrogenesis [7]. Heterozygous mutations of Sox9 were shown to cause the severe skeletal malformation syndrome and completely absence of Sox9 can block chondrogenesis [8]. Furthermore, Sox9 underlies chondrocyte differentiation through transcriptional activation of many genes that are essential to build and maintain health cartilage. And many other genes play the role of regulating chondrocyte differentiation probably through collaborating with Sox9 or affecting Sox9 expression [6]. Sox5 and Sox6, two members of the SOX family, can activate chondrocyte differentiation through coexpression with Sox9 [9, 10]. However, although upregulation of Sox9 can promote chondrogenic differentiation of MSCs, it also slows the process of chondrocyte hypertrophy via affecting some key genes expression [11, 12]. Bi et al. reported that the loss of Sox9 changed immature chondrocytes into hypertrophic cells [13]. All these studies suggested that Sox9 is required to establish the chondrogenic lineage.

Sox9 represses chondrocyte hypertrophy mainly through several ways as follows: (1) It can block the activation of the transcription factor Runx2, which is the key factor in reducing chondrocyte maturation [14]; (2) Topol et al. reported that Sox9 interacted with *β*-catenin to inhibit Wnt signaling which has been demonstrated to promote chondrocyte hypertrophy [15]; (3) Sox9 may directly repress expression of the genes that are expressed in hypertrophic chondrocytes, such as Col10a1 and VEGFA [16, 17].

## 3. Bone Morphogenetic Protein Signaling

BMPs were identified to be a positive regulator in ectopic chondrogenesis and endochondral ossification [18, 19]. It is reported that inhibition of BMP signaling will suppress the formation of cartilage [20]. BMP signaling is mediated by their receptors BMPR1 (BMPR1a and BMPR1b) and BMPR2 [21, 22]. Activation of these receptors leads to the phosphorylation of SMAD transcription factors such as SMAD1, SMAD5, and SMAD8, which are demonstrated to regulate the expression of target genes in early xenopus embryos [23]. Inversely, a knockout of each of these receptors in mice displays a lack of expression of Sox5, Sox6, and Sox9 in precartilaginous condensations, so as to inhibit chondrocyte formation and result in defective maturation of chondrocytes [22, 24].

## 4. Wnt Signaling

Wnt signaling is essential for many developmental processes, including the skeletogenous process. Chondrocyte and osteoblast are two primary cell types in the skeletal systems that are differentiated from common mesenchymal progenitors. It is shown that Wnt signaling can regulate chondrocyte and osteoblast differentiation of the chondroosseous progenitor cells. Activation of Wnt signaling promotes osteoblast differentiation but suppresses chondrocyte differentiation of MSCs [25, 26]. Wnt can be divided into two classes: Wnt-1 class (Wnt-1, -3a, -7a, and -8, etc.), which activates the canonical Wnt pathway and Wnt-5a class (Wnt-4, -5a, and -11, etc.), which activates the noncanonical Wnt pathway [27]. Canonical Wnt signaling acts through *β*-catenin to promote chondrocyte hypertrophy and reports suggested that genetic inactivation of *β*-catenin increased Sox9 expression and induced chondrocyte differentiation at the expense of osteoblast differentiation both in the process of intramembranous and in endochondral ossification [28, 29]. What is more, osteoblast precursors lacking *β*-catenin are demonstrated to develop into chondrocytes instead [25]. Reinhold et al. showed that Wnt3a strongly repressed chondrogenesis and chondrocyte gene expression [26]. There are also some other studies about the noncanonical Wnt signaling. Liu et al. suggested that Wnt11 overexpression stimulated the gene expression of chondrogenic regulators and promoted chondrogenic differentiation of MSCs in synergism with TGF-*β* [30]. Yang et al. indicated that Wnt5a and Wnt5b appear to coordinate chondrocyte proliferation and differentiation by regulating chondrocyte-specific Col2a1 expression [31]. Taken together, these findings suggest that Wnt signals acting via *β*-catenin seem to block chondrocyte differentiation but to promote chondrocyte hypertrophy and that the effects of noncanonical Wnt signals in chondrocyte formation and maturation are different from canonical Wnt signals.

Wnt signaling also plays an important role in regulating the proper orientation of chondrocyte columns in the growth plate. It has been reported that Wnt5a and Wnt5b seem to regulate the region of proliferating chondrocytes, which locate at the distal ends of the condensation. The proliferation rate and region of proliferating chondrocytes were reduced in Wnt5a-deficient mice [31]. Bradley and Drissi indicated that Wnt5b regulated mesenchymal cell aggregation and chondrocyte differentiation through activating the Wnt planar cell polarity pathway [32]. Disrupting Wnt planar cell polarity pathway in vivo results in loss of columnar growth plate architecture, and activation of this pathway in chondrocyte cell pellets promotes columnar organization in these cells that are normally oriented randomly in culture. Randall et al. showed that activation of Wnt planar cell polarity pathway in the presence of recombinant Wnt5a or Wnt5b promoted the initiation of columnar morphogenesis in the chondrocyte pellet culture model [33].

## 5. Fibroblast Growth Factor Signaling

Fibroblast growth factors (FGFs) are members of a large family of signaling proteins. The mutations in FGFs and their receptors have been defined with essential roles for FGF signaling in both endochondral and intramembranous bone development. It has proved to be important in the regulation of chondrocyte proliferation and the initiation of chondrocyte hypertrophy. Fibroblast growth factor receptor 1 (FGFR1) and FGFR2 are both expressed in condensing mesenchyme that will differentiate into cartilage. FGFR1 is expressed in hypertrophic chondrocyte and FGFR1 deficiency leads to a transient increase in the hypertrophic zone [34]. FGFR2 is initially expressed at high levels in condensing mesenchyme and appears to be downregulated in proliferation chondrocyte [35]. Furthermore, the loss of FGFR2 results in postnatal dwarfism with reduced thickness of the hypertrophic zone [36]. FGFR3 is expressed in proliferating chondrocytes to regulate cell growth and differentiation and is downregulated in the hypertrophic zone [37]. Activating mutations in FGFR3 inhibits chondrocyte proliferation and the initiation of chondrocyte hypertrophy but accelerates late hypertrophic differentiation [37, 38].

Although the FGF ligands involved in skeletal development have been well characterized, only FGF9 and FGF18 have been shown to be relative to chondrogenesis. FGF9 both directly and indirectly promotes chondrocyte proliferation and hypertrophy at early stages and regulates vascularization at later stages in endochondral ossification [34, 39]. In FGF18 signaling, it promotes both proliferation and the initiation of chondrocyte hypertrophy during early stage, and it blocks chondrocyte proliferation and delays chondrocyte hypertrophy in older embryos [39–41].

## 6. Indian Hedgehog Signaling and Parathyroid Hormone-Related Peptide Signaling

Ihh is a key regulator of endochondral ossification, which is expressed and secreted by prehypertrophic and early hypertrophic cells. Ihh signaling directly activates proliferation in proliferating chondrocytes and Ihh-deficient mice display a markedly reduced chondrocyte proliferation, premature chondrocyte hypertrophy, and a failure of osteoblast development in endochondral bones [42]. Furthermore, Ihh controls the expression of PTHrP. Overexpression of PTHrP results in delayed chondrocyte differentiation and the deletion of PTHrP causes diminished chondrocyte proliferation, maturation of chondrocytes at inappropriate position, and accelerated bone formation [43]. It has been proved that the inhibition of chondrocyte hypertrophy by increased Ihh could be abolished by the deletion of PTHrP [44]. Studies indicate that activation of Ihh signaling upregulates PTHrP and prevents chondrocyte hypertrophy but not PTHrP null explants, suggesting that Ihh regulates chondrocyte proliferation and maturation by a PTHrP-dependent pathway [45]. What is more, there is also a PTHrP-independent pathway that positively regulates chondrocyte proliferation mainly through the transcription factors of the GLI family [45, 46].

## 7. Runx Family Transcription Factors

Runx2 and Runx3 are members of the Runx family transcription factors that are important for promoting chondrocyte hypertrophy. Several studies demonstrate that the ectopic expression of Runx2 in immature chondrocytes leads to the expression of hypertrophic markers such as Col10a1, MMP13, and VEGF [47–49]. It is found that Runx2 can interact with BMP-regulated Smads proteins to activate hypertrophic chondrocyte gene expression and directly bound to the promoter region of the Ihh gene to strongly induced expression of the reporter gene driven by the Ihh promoter [50, 51]. A knockout of Runx2 in mice results in delayed and markedly reduced chondrocyte hypertrophy and the Runx3-deficinent mice show a slight delay in chondrocyte hypertrophy and vascular invasion into cartilage [50, 52]. What is more, the knockout of both Runx2 and Runx3 in animals showed a complete absence of chondrocyte maturation [50]. All these findings indicate that Runx2 and Runx3 are essential for chondrocyte maturation.

## 8. Adenosine Signaling

Purines are important cellular metabolites, which are involved in a wide variety of processes. Adenosine is a nucleoside that can be generated by enzymatic degradation of adenine nucleotides both intracellularly and extracellularly [53–55]. Increase of extracellular adenosine levels leads to increased intracellular adenosine concentration via transporters. Adenosine has an important physiological signaling role in the peripheral and central nervous systems and it is known to take part in several different metabolic pathways by activating cell surface G protein-coupled receptors [56]. However, abnormal levels of adenosine may be bad for health [57, 58]. A degradative pathway converts adenosine to inosine which is mediated by adenosine deaminase (ADA). Deficiency of adenosine deaminase will affect the skeletal, central nervous, endocrine, and gastrointestinal systems [59, 60]. The second pathway is to convert adenosine to AMP by adenosine kinase (AK) and then is further metabolized into adenosine triphosphate (ATP) and dATP [61]. Keeping the balance between ATP and dATP is important in effective DNA repair. There are four adenosine receptors among vertebrates named A1, A2A, A2B, and A3, which are divided into two subclasses: negatively coupled to (A1 and A3) or stimulate (A2A and A2B) adenylate cyclase [55, 59, 60]. However, unlike A2B with a low affinity, A2A has a high affinity [62]. What is more, these receptors can impact other signaling pathways such as mitogen-activated protein kinases (MAPKs) and serine-threonine-specific kinases to affect multiple systems [63].

In recent years, studies indicated that adenosine plays an indispensable role in bone and cartilage development. ADA-deficient patients often present myeloid dysplasia features and bone marrow hypocellularity [64]. Manson et al. showed that unusual chondroosseous findings could be seen in patients with ADA deficiency and these anomalies would be resolved during 6–12 months of ADA enzyme replacement therapy when adenosine levels are back to normal [65]. ATP as a Ca^2+^ modulator was found to maintain proteoglycan values close to native tissue and increase collagen synthesis and functional properties of engineered cartilage by increasing Ca^2+^ oscillations in monolayer-cultured chondrocytes [66]. Study in MC615 chondrocyte demonstrates that increasing adenosine concentrations within the chondrocytes and blocking its degradation by an ADA inhibitor (erythro-9-(2-hydroxy-3-nonyl) adenine (EHNA)) will cause cell death [61].

Chondrocytes have ability to release adenosine upon a variety of physiological stimuli. Both the extracellular and endogenous adenosine levels have an important role of regulating cartilage damage during inflammatory processes. If the levels drop, the expression of glycosaminoglycans, matrix metalloproteinases (MMP3, MMP13), and nitric oxide (NO) will increase, thereby promoting chondrogenic differentiation [61]. However, if the chondrocytes are incubated with ADA inhibitor, the higher adenosine concentration leads to reduction of GAG, NO, and prostaglandin E2 release, so as to cause cartilage damage but reduce inflammation [67]. What is more, the stimulation of adenosine receptor A2AR reduced proinflammatory cytokines and production of MMPs by inhibiting NF-kB activation in articular chondrocytes of mouse which is previously stimulated with interleukin-1*β* [68–70]. Therefore, adenosine may have a dual role in cartilage damage and there may be a role for activating A2AR to prevent bone and cartilage erosion in rheumatoid arthritis.

## 9. Role of Oxygen Tension in Chondrocyte Development

Articular cartilage is an avascular tissue and its nutrition is supplied mainly by synovial fluid and partly from subchondral bone [71, 72]. Oxygen (O_2_) levels within the tissue have been described to be between 1% and 6% [73, 74]. Subsequently, chondrocytes have to be adapted to a low O_2_ tension environment. However, O_2_ tension below 1% will inhibit glucose uptake, lactate production, and cellular ribonucleic acid (RNA) synthesis [75]. In other words, at least some O_2_ is needed for the basal metabolism of chondrocytes. Studies indicate that both anoxia and hyperoxia are harmful to chondrocytes via impacting glycolysis and matrix production [76–79].

How does O_2_ modulate chondrocyte activities? It is apparent that cell survives better at 5% than that at 20% O_2_ for a cell originating from tissue with low oxygen tension [80]. The shape of chondrocytes will become similar to spindle-like phenotype at a high O_2_ tension environment [81, 82]. Synthesis of extracellular matrix components including collagen type II and glycosaminoglycan is greater under hypoxic (5%) condition than that under normoxia (20%), either in quality or in quantity [76, 83, 84]. O_2_ tension also has effect on chondrocyte gene expression. Sox9, collagen II, and aggrecan that are used to characterize the chondrogenic induction are strongly suppressed at high O_2_ tension [85, 86]. Bovine chondrocytes show a higher collagen type II expression and a lower collagen type I expression under hypoxia compared to normoxia [87, 88]. And the normal articular cartilage collagen subtypes II, IX, and XI are converted to collagen I, III, and V at high O_2_ level [85, 89, 90]. What is more, low O_2_ tension can stimulate chondrocytes redifferentiation of dedifferentiated chondrocytes with reexpression of collagen II and GAGs [85, 88, 91].

It is also demonstrated that hypoxia can stimulate MSCs to differentiate into chondrocytes [86, 92–94]. Hypoxia inducible factors (HIFs) are a group of critical transcription factors which mediate cellular responses to changes of oxygen tension [95]; the stability and transactivation of HIFs are essential to the effect of hypoxia on the chondrogenic differentiation of MSCs. Under normoxic conditions, HIF-1*α* usually exists in the cytoplasm instantaneity and is transitorily decomposed by ubiquitin-proteasome pathway. HIF-1*α* will translocate to the nucleus to bind its DNA binding site to arouse the expression of hypoxia-related genes under hypoxia [95, 96]. Studies suggest that expression of HIF-2*α* was upregulated under hypoxic chondrogenic induction compared to that under normoxic chondrogenic induction [97, 98]. Except for HIFs, PI3K/Akt/FoxO pathways may also be involved in enhanced chondrogenic differentiation under hypoxia by inhibiting apoptosis [99]. In Portron et al.'s study, low oxygen tension (5% O_2_) not only stimulates the early chondrogenic differentiation of human adipose MSC and murine ATDC5 cells, but also inhibits their hypertrophic differentiation by altering the transcriptional activity of HIF-1*α* and HIF-2*α* [100]. Additionally, a concentration gradient of oxygen tension can direct the chondrogenic differentiation of human MSC into either permanent cartilage or hypertrophic cartilage that is destined to be replaced by bone [101]. Hypoxia (2.5% O_2_) promoted human MSCs chondrogenic differentiation and normoxia (21% O_2_) increased hypertrophic differentiation [101].

These studies indicate that, although chondrocytes are well adapted to hypoxia, chondrocyte phenotype is sensitive to O_2_ tension. Low O_2_ tension promotes the cartilage-specific matrix formation and the expression of the chondrogenic phenotype, suggesting that O_2_ tension has a very important role in chondrogenesis and cartilage degradation, and it may be a key factor in cartilage tissue engineering and stem cell therapy.

## 10. Role of Reactive Oxygen Species in Chondrocyte Development

It is suggested that the effects of O_2_ on cartilage are mediated partly through reactive oxygen species (ROS) [102]. Oxygen can be processed into reactive oxygen species (ROS) under some physiological and pathological stimuli. In order to respond to the partial oxygen pressure variations, chondrocytes produce abnormal levels of ROS to resist stress [103, 104]. And it is reported that low levels of ROS can act as one of the intracellular second messenger to regulate various of radical cellular activities, such as cell activation, proliferation, and death, and also mediate the expression of a series of genes [105–108]. However, excessive levels of ROS can cause pathological conditions such as inflammatory joint diseases [109, 110]. ROS produced by cells are molecules like hydrogen peroxide (H_2_O_2_), radicals like nitric oxide (^•^NO), hydroxyl radical (^•^OH), and the superoxide anion (O_2_^−^) which is an ion and a radical at the same time and can generate derivative radicals peroxynitrite (ONOO^−^) [107, 109].

**Figure 1:**
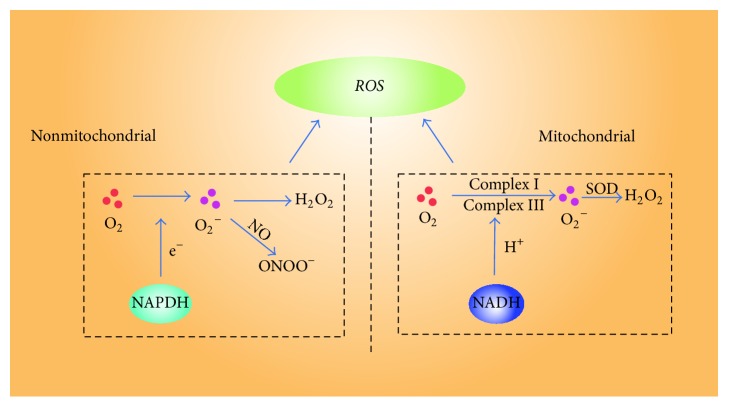
The main sources of ROS in chondrocytes. ONOO^−^ = peroxynitrite; O_2_^−^ = superoxide anions; H_2_O_2_ = hydrogen peroxide.

The generation of ROS can be physiological, pathological, and tissue specific according to different situations [111]. It always exists in mitochondria, peroxisomes, endoplasmic reticulum, cytosol, the plasma membrane, and the extracellular space [112–114]. There are two ways to generate ROS; first is through mitochondrial electron transport systems by NADPH oxidases, xanthine oxidase, nitric oxide synthases, lipoxygenases, and so forth [115, 116]. Second is called nonmitochondrial ROS production that is mainly produced by NOX enzymes and NADPH oxidases [114] (Figure 1). NOX enzymes reduce oxygen to superoxide by the pyridine nucleotide NADPH as an electron donor and molecular oxygen as electron acceptor. ROS is generated as the secondary product.

It has been provided that ROS was involved in the activity of articular chondrocytes as signaling intermediates for cytokines and growth factors. The expression of collagenase induced by IL-1*β* in chondrocytes was found to be ROS-dependent [106]. Inhibiting the production of ^•^NO partially reduced the IL-1 induction of collagenase expression and treating chondrocytes with ^•^NO is able to stimulate collagenase gene expression [106]. That is, the responses of cells to cytokines and growth factors are relied on the redox status. Thus, the balance between ROS and intracellular antioxidants level is important for us to understand the role of ROS in the homeostasis of cartilage tissue and physiopathology of arthritis.

### 10.1. Chondrocyte Apoptosis

Chondrocytes death is an important factor to break down extracellular matrix in joint disease. Chondrocyte apoptosis is related to ^•^NO which is considered as the primary inducer mediated by caspase-3 and tyrosine kinase activation [117]. It is reported that resveratrol prevents sodium nitroprusside-induced chondrocyte apoptosis via scavenging ROS [118]. Lee and Yang demonstrated that polychlorinated biphenyl 126 is an initiator of chondrocyte apoptosis via generating ROS, increasing NO production and NF-kB binding activity in the chondrocytes [119]. However, some studies suggested the apoptotic effect of ^•^NO could not be realized by itself; there should be a role for other ROS such as O_2_^−^ or ONOO^−^ in this process [120, 121].

Inversely, Del Carlo Jr and Loeser have proposed that ^•^NO can be antiapoptotic when the intracellular antioxidant is very low [120]. This is probably because hypochlorous acid reacts with NO_2_^−^ to produce nitryl chloride and nitryl chloride is less damaging to cells than hypochlorous acid. Therefore, both hypochlorous acid and ^•^NO are overproduced which can be carried out to protect cell against damage caused by the hypochlorous acid. And there may be another mechanism for ^•^NO to be antiapoptotic by inhibiting the activation of Fas-induced caspase-3 [122]. In conclusion, ROS may play an important role in chondrocyte apoptosis [123].

### 10.2. Matrix Degradation

In vitro and in vivo studies showed that ROS played an important role in cartilage matrix degradation, mainly reflected on the effect of matrix composition and chondrocyte behavior. Studies indicated that ROS contributes to cartilage degradation via activating the collagenase and upregulating the expression of encoding gene of MMPs. What is more, ROS inhibitor and ROS scavengers can slow down the loss of cartilage. Reed et al. showed the increase in ROS led to an increase in MMPs levels which is the main component of degrading cartilage matrix, and there was a subsequent reduction in MMP levels with ROS scavenger added. It reveals that ROS is essential for maintaining the cartilage matrix by altering the level of MMP [124, 125]. Shingu et al. suggested IL-6 inhibited superoxide production in chondrocytes and thus inhibited cartilage matrix degradation, and oxygen radical-mediated activation of collagenase in chondrocytes can explain how oxygen radicals are involved in the mechanisms of cartilage matrix degradation [126]. ROS may cause damage to all matrix components by directly attacking or indirectly reducing the synthesis of cartilage matrix components, by inhibiting growth factor bioactivity, by inducing chondrocytes apoptosis, or by activating latent metalloproteinases [109, 127]. These findings support the possibility of using antioxidants for the treatment of rheumatic diseases.

There are many intracellular signaling pathways regulated by intracellular ROS in chondrocyte differentiation such as phosphatidylinositol 3-kinase/Akt (PI3K/Akt) and mitogen-regulated kinase (MAPK) pathways, which have been reported to play an important role in the differentiation of cartilage [109]. It has been indicated that the thymoquinone-induced production of ROS regulates chondrocyte apoptosis by modulating PI3K/Akt and p38 kinase pathways, which causes rabbit articular chondrocytes dedifferentiation through the ERK pathway and inflammation through the PI3K and p38 pathways [128, 129].

ROS plays crucial roles in the regulation of some normal chondrocyte activities. Evidence has been provided that as long as ROS production is under the control of the cellular antioxidant in articular chondrocytes, it can play great role as integral actors of cartilage homeostasis and intracellular signaling mechanisms. However, once ROS is produced in greater amounts so as to exceed the antioxidant capacities of the cell, it will contribute to cartilage damage in structure and function. The regulation of ROS in cartilage is a complex process. Further research is needed to unravel the relationship between ROS generation and chondrocyte metabolism, which would be a major discovery in the treatment of articular cartilage disease and prevention of cartilage aging.

## 11. Other Factors Involved in Chondrogenesis

**Figure 2:**
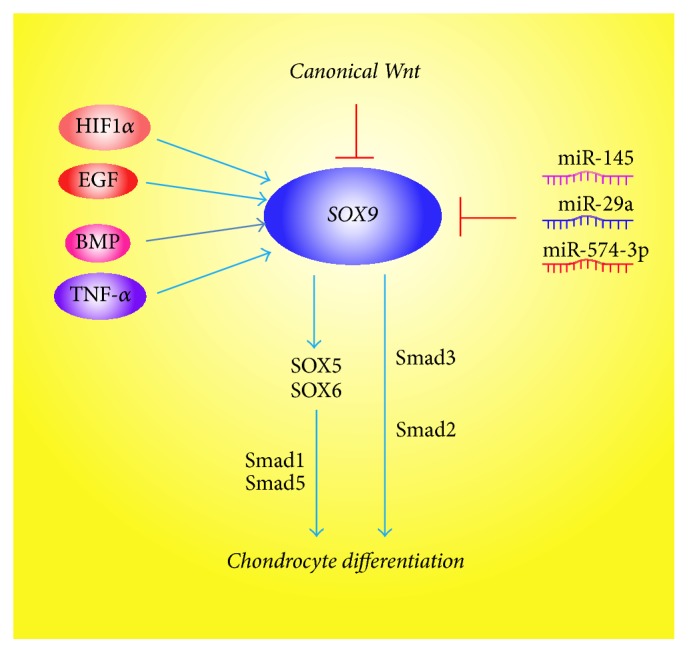
Multiple signaling pathways regulate the chondrocyte differentiation through regulating Sox9 expression and activity.

**Figure 3:**
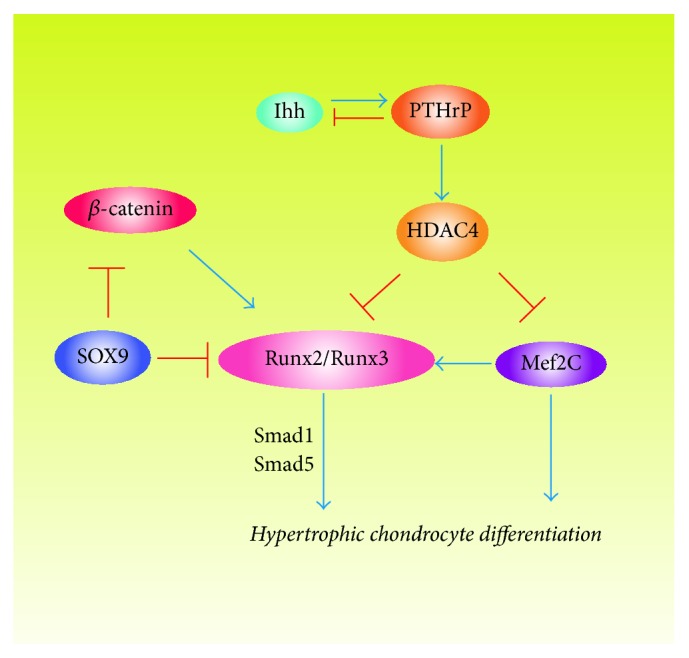
Different combinations of transcription factors regulate hypertrophic chondrocyte differentiation.

There are also some other factors involved in the differentiation of MSCs to chondrocyte and hypertrophic chondrocyte (summarized in Figures 2-3). For example, the catalytic subunit of cyclic AMP- (cAMP-) dependent protein kinase A increases the activity of Sox9 and the inhibition of protein kinase A blocks the chondrocyte differentiation [130, 131]. Retinoic acid signaling represses Sox9 expression and blocks chondrocyte differentiation partly by promoting canonical Wnt signaling [132]. Mef2c is a member of the myocyte enhancer factor 2 families, which is expressed in prehypertrophic and hypertrophic chondrocytes. The loss of Mef2c leads to downregulation of Runx2 expression and a delay in chondrocyte hypertrophy because the Mef2c functions upstream Runx2 [133]. TNF-*α* was also reported to increase the expression of Sox9 to promote the chondrocyte differentiation of MSCs [134].

In addition, chondrocyte differentiation is also regulated by some microRNAs via altering the expression of Sox9. It has been shown that miR-145 is a key negative regulator of chondrogenic differentiation by directly targeting Sox9 to regulate the mRNA levels of chondrogenic marker genes and it contributes to impaired extracellular matrix in osteoarthritis partly via targeting Smad3 [12, 135, 136]. The upregulating of miR-574-3p results in inhibition of Sox9 expression and chondrogenesis [137]. Guérit et al. demonstrated that downregulation of miR-29a was essential in chondrocyte differentiation of MSCs [138].

## 12. The Ultimate Fate of Hypertrophic Chondrocyte

Although there are so many signaling factors that have been indicated to be regulators of both chondrocyte proliferation and chondrocyte hypertrophy, the ultimate fate of hypertrophic chondrocyte is still controversial. The current dogma suggests that chondrocytes and osteoblasts are independent lineages derived from a common osteochondroprogenitor and it is generally accepted that cell apoptosis is the fate of late hypertrophic chondrocyte in endochondral bone formation. However, in recent study, Yang et al. used a cell-specific tamoxifen-inducible genetic recombination approach to track the fate of murine terminally differentiated hypertrophic chondrocytes and found that a part of the hypertrophic chondrocytes undergo death, while a considerable number of these cells still existed in cartilage-to-bone transition and became osteoblasts and osteocytes [139]. In other words, the ultimate fate of hypertrophic chondrocyte is not yet well-understood and further research should be carried out for chondrocyte-based and/or stem cell-based strategies of cartilage regeneration.

## 13. Conclusion

Chondrocyte differentiation is regulated by multiple signal transduction pathways, which form a complex transcriptional network. During the last decades, great advances have been made in the analysis of the complex transcriptional network that regulates chondrocyte differentiation. The balance of these signaling pathways is essential for normal chondrocyte differentiation and cartilage development. Better understanding of these signaling pathways will help us comprehend the process of chondrogenesis and vertebrate development, which is necessary in the regulation of cartilage repair and bone repair.
